# Longitudinal Associations Between Physical Activity and Sedentary Time and Cardiorespiratory and Muscular Fitness in Preschoolers

**DOI:** 10.3390/jfmk9040199

**Published:** 2024-10-21

**Authors:** Kirkke Reisberg, Eva-Maria Riso, Liina Animägi, Jaak Jürimäe

**Affiliations:** 1 Department of Physiotherapy and Environmental Health, Tartu Health Care College, 50411 Tartu, Estonia; kirkkereisberg@nooruse.ee (K.R.); liinaanimagi@nooruse.ee (L.A.); 2 Institute of Sport Sciences and Physiotherapy, Faculty of Medicine, University of Tartu, 51008 Tartu, Estonia; eva-maria.riso@ut.ee

**Keywords:** physical activity, sedentary time, cardiorespiratory fitness, muscular fitness, children

## Abstract

**Background/Objectives:** The impact of physical activity (PA) and sedentary time (ST) during preschool years on the physical fitness (PF) levels of school-aged children remains unaddressed. This study aimed to investigate the associations of objectively measured vigorous physical activity (VPA), moderate-to-vigorous physical activity (MVPA), total physical activity (TPA), and ST in the last year of preschool (age of 6–7 years; *n* = 77; 51% boys) with cardiorespiratory fitness (CRF) and muscular fitness (MF) in the first grade of school among Estonian children. **Methods:** We assessed PA (accelerometers), CRF (20 m shuttle run), and MF (z-score of relative upper- and lower-limb muscular strength). **Results:** In the unadjusted analysis, higher VPA, MVPA, and TPA in preschool were associated with a higher MF in school among boys, while a higher VPA in preschool was related to a higher CRF in school among girls. However, VPA, MVPA, TPA, and ST in preschool were unrelated to CRF and MF among boys and girls after adjustment for baseline age, accelerometer wear time, the corresponding PF item, and parent’s education. In addition, a higher PF level in preschool was frequently related to a higher corresponding PF item in school among both genders. **Conclusions:** Moderate-to-vigorous and vigorous type of activities during final year of preschool, as well the amount of TPA that preschoolers are involved in, are not sufficient to affect their CRF and MF longitudinally. In addition, ST in preschool did not impact the CRF and MF of boys and girls in the first grade.

## 1. Introduction

Physical fitness (PF) is the capacity to perform physical activity (PA), and it makes reference to a full range of physiological and psychological qualities [1].

More specifically, cardiorespiratory fitness (CRF) represents the overall capacity of the cardiovascular and respiratory systems, and the ability to perform prolonged strenuous exercise [1]. It has been proven that performance on a 20 m shuttle run is associated with multiple health indicators among children [2]. On the other hand, low levels of CRF at a young age have been attributed to a higher risk of developing cardiovascular disease in adulthood [3]. Muscular fitness (MF) is the ability to perform work against a resistance [1]. Relative handgrip strength at 5–10 years of age was positively related to different PF parameters, such as absolute handgrip strength, vertical jump, peak oxygen consumption, and performance in a 20 m shuttle run test [4], suggesting that a high upper-limb strength expresses the general fitness of a young person. Relative handgrip strength was inversely associated with metabolic syndrome, insulin resistance [5], present and future cardiovascular disease risk [6], and ideal cardiovascular health [7] among children and adolescents. As for upper-limb strength, a better performance on the standing long jump (SLJ) test predicted lower cardiovascular disease risk for children and adolescents [6] and was a determinant of healthier bone structure and higher strength at preschool age [8]. Also, SLJ was negatively associated with negative affect and positively with self-esteem among preadolescents [9].

Muscular fitness compound measures have also been used in studies to express overall MF [10,11,12]. Research shows that MF has a beneficial impact on bone health in children and adolescents with overweight and obesity [10,11]. Also, a positive association of MF with cognitive abilities among preadolescents was found [12]. Accordingly, it appears that PF is a notable indicator of health status already at an early age [1].

Cross-sectional studies among preschool aged children demonstrate that a higher vigorous PA (VPA) [13,14,15], moderate-to-vigorous PA (MVPA) [14,15], and total PA (TPA) [13] were beneficial for CRF, and higher VPA and MVPA were associated with a higher lower-limb strength [14,15]. At the same time, while Leppänen et al. [14] showed that VPA and MVPA were related to a higher upper-limb strength, another study detected no associations with upper-limb strength [15]. Regarding longitudinal associations among preschoolers, it is shown that a higher VPA and MVPA were associated with a higher CRF in one study [16], yet only VPA, but not moderate PA (MPA) or TPA, were related to the improvement in CRF during the follow-up period in another study [13]. With respect to muscular strength, a higher VPA and MVPA were longitudinally associated with a higher lower-limb strength, but not with upper-limb muscular strength [16]. To sum up, there is a lack of studies investigating the associations between PA and PF among preschool aged children. Considering that PA is the major determinant of PF [13], the relationships between PA and PF during preschool years need to be explored in order to understand and potentially improve healthy lifestyle solutions.

Findings about the cross-sectional associations between time spent sedentary with CRF and muscular strength during preschool years are mixed. In one study, a higher sedentary time (ST) was related to a lower CRF [15], while no associations were found in another study [14]. In one study, ST was negatively associated with upper-limb strength after adjustment for several confounding factors, but after adjusting also for VPA, the association disappeared [14]. In another study, ST was not associated with upper-limb strength [15]. Regarding lower-limb strength, it has been found that a higher ST was related to lower lower-limb strength after adjustment for age and gender, but the associations disappeared when the data were additionally adjusted for organized sport participation and parents’ education level [15]. Leppänen et al. [14] found ST and lower-limb strength to be unrelated. Longitudinally, ST at the age of 4.5 years did not predict CRF or upper- and lower-limb muscular strength [16]. Thus, in terms of studies about the associations between PA and PF, there is not much research for the associations between time spent being sedentary and PF among preschool-aged children. In view of the fact that, already in preschool, children spend the majority of the time in sedentary activities [17,18], with a potential impact on their physical capacity and therefore on their health, the associations between ST and PF among preschoolers should be further explored.

As boys and girls spend different amounts of time conducting PA [19], as well their PF [20,21] being different, it is important to investigate the relationships between PA, ST, and PF among both genders separately. Hence, the specific aim of this study was to investigate whether VPA, MVPA, TPA, and ST during preschool are associated with CRF and MF in the first grade of school in boys and girls.

## 2. Materials and Methods

### 2.1. Participants

A total of 13 kindergartens in Estonia were recruited in the study. From 400 children and their parents, 284 volunteered to participate in the assessments performed when children were in their last year of kindergarten. A year later, children (*n* = 200) were measured again in the school.

Baseline data from 82 children (51.2% boys) were included in this analysis. Data on the associations of VPA, MVPA, TPA, and ST in preschool with CRF and MF in school were applicable for 41 boys and 36 girls.

The education levels of the parents were graded as basic, general secondary/vocational, or higher and the data of higher-educated parents were included. The body height of the participants was measured using a portable stadiometer (Seca 213, Hamburg, Germany), and a digital medical scale (A&D Instruments, Abington, UK) was used to measure the body weight as described in our previous study [22].

Approval from the Research Ethics Committee of the University of Tartu (references 254/T-13 and 266/T-8) was obtained. The study is compatible with the ethical standards of the Declaration of Helsinki.

### 2.2. Assessment of Physical Activity

To measure PA and ST, the triaxial Actigraph GT3X accelerometer (ActiGraph LLC, Pensacola, FL, USA) was worn on the hip for seven days in a row during waking hours, excluding during for bathing and swimming [23]. The data were collected at intervals of 15 s. The participants with at least three days (incl. one weekend day) of ≥10 h daily accelerometer wear time (AWT) were included in the analysis [24]. Non-wearing time was characterized as ≥20 min periods of consecutive readings of zero counts and was excluded from the analysis [25]. ST was defined as the time spent with exertion levels below 100 counts per minute. Counts of 100 to 1999, 2000 to 3999, and ≥4000 per min differentiated light PA (LPA), MPA, and VPA, respectively [26,27]. We calculated the average time per day (min/day) spent in each intensity zone as follows: (weekdays*5 + weekends*2)/7 [28]. MVPA was calculated by summing the time spent in MPA and VPA [29,30]. TPA was determined by summing up LPA, MPA, and VPA [31]. AWT was calculated by the summing of ST, LPA, MPA, and VPA [16]. 

### 2.3. Assessment of Physical Fitness

A 20 m shuttle run test was used to assess CRF [32]. The children were asked to run back and forth over a distance of 20 m. The test begun at a speed of 8.5 km/h, which was increased every minute by 0.5 km/h. The test ended when the children could not follow the set pace of the test. The number of laps completed was recorded [33].

Lower-limb muscular strength was assessed by the SLJ test (cm). From the starting line, jumping forward as far as possible was performed [34]. The best result of two attempts was taken into account [35].

Upper-limb muscular strength was assessed by a digital dynamometer (Digital TKK 5401, Grip D, Takei, Tokyo, Japan). The children squeezed the dynamometer continuously for 2–3 s. Two attempts with each hand were performed, and the best result was taken into account. The mean of the left and right handgrip strength (kg) was calculated [36] and expressed relative to the body mass (in kg/kg) [10].

Muscular fitness z-score was generated from the mean of gender-specific z-scores of the relative upper- and lower-limb muscular strength and has been introduced in other studies [10,37].

### 2.4. Statistical Analysis

Statistical analysis was conducted with the SPSS software (version 20.0; SPSS, Inc., Chicago, IL, USA). A *p*-value of less than 0.05 was considered statistically significant. The data from boys and girls were analysed separately. Continuous variables were assessed for normality by the Shapiro–Wilk test. Continuous variables that were normally distributed were compared by a Student’s *t*-test, and the Mann–Whitney U test was used for skewed variables.

By multiple linear regression analysis, the associations of VPA, MVPA, TPA, and ST in preschool with the CRF and MF z-score in school were examined; baseline values of exposures (VPA, MVPA, TPA, and ST) and the baseline value of outcome (CRF or MF z-score) [38] were included in the adjusted model. Age [16,27,38,39], parents’ education [38,40,41,42], and AWT [16,39,43,44] at baseline were added to the adjusted model. 

## 3. Results

### 3.1. Characteristics of the Study Population at Baseline

Boys had a higher [26 (6) vs. 23.5 (6) kg] (*p* = 0.010) median weight than girls. Boys were also taller (127 ± 5.4 vs. 124 ± 6.4 cm) (*p* < 0.001) compared to girls. Boys had a higher median VPA [18 (12.7) vs. 13.5 (12.3) min/day] (*p* = 0.042), median MVPA [62.9 (29.4) vs. 57.6 (29.2) min/day] (*p* = 0.003), and TPA [382 ± 59.2 vs. 359 ± 41.5 min/day] (*p* = 0.001) than girls. Boys showed a better median handgrip strength [11.5 (3) vs. 9.9 (2.7) kg] (*p* = 0.007) than girls (Table 1). The parents’ education level was similar among both genders (*p* = 0.765).

### 3.2. Associations of VPA, MVPA, TPA, and ST in Preschool with CRF in School

Among boys, VPA in preschool was not associated with CRF in school. CRF in preschool was positively associated with CFR in school (*p* < 0.001) after adjusting for the confounding factors (R^2^ = 0.402, F = 4.572, *p* = 0.003). Among girls, a higher VPA (R^2^ = 0.106, *p* = 0.038) in preschool was associated with a higher CRF in school. We found no associations after adjustment for the confounding factors (F = 1.499) (Table 2).

Among boys, MVPA in preschool was not associated with CRF in school. CRF in preschool was positively associated with CFR in school (*p* < 0.001) after adjusting for the confounding factors (R^2^ = 0.303, F = 4.387, *p* = 0.003). Among girls, MVPA in preschool was not associated with CRF in school. We found no associations in the adjusted model (F = 1.590) (Table 2).

Among boys, TPA in preschool was not associated with CRF in school. CRF in preschool was positively associated with CFR in school (*p* < 0.001) after adjusting for the confounding factors (R^2^ = 0.315, F = 4.590, *p* = 0.003). Among girls, TPA in preschool was not associated with CRF in school. We found no associations after adjustment for the confounding factors (F = 1.498) (Table 2).

In boys, ST in preschool was not associated with CRF in school. CRF in preschool was positively associated with CRF in school (*p* < 0.001) after adjusting for the confounding factors (R^2^ = 0.315, F = 4.590, *p* = 0.003). In girls, ST in preschool was not associated with CRF in school. We found no associations after adjustment for the confounding factors (F = 1.498) (Table 2).

### 3.3. Associations of VPA, MVPA, TPA, and ST in Preschool with Muscular Fitness in School

In boys, a higher VPA (R^2^ = 0.119, *p* = 0.015) in preschool was associated with a higher MF in school. MF in preschool was positively associated with MF in school (*p* < 0.001) after adjusting for the confounding factors (R^2^ = 0.457, F = 7.554, *p* < 0.001). In girls, VPA in preschool was not associated with MF in school. MF in preschool was positively associated with MF in school (*p* = 0.001) after adjusting for the confounding factors (R^2^ = 0.426, F = 6.046, *p* = 0.001) (Table 2).

In boys, a higher MVPA (R^2^ = 0.119, *p* = 0.015) in preschool was associated with a higher MF in school. MF in preschool was positively associated with MF in school (*p* < 0.001) after adjusting for the confounding factors (R^2^ = 0.461, F = 7.660, *p* < 0.001). In girls, MVPA in preschool was not associated with MF in school. MF in preschool was positively associated with MF in school (*p* = 0.001) after adjusting for the confounding factors (R^2^ = 0.430, F = 6.123, *p* = 0.001) (Table 2).

In boys, a higher TPA (R^2^ = 0.113, *p* = 0.018) in preschool was associated with a higher MF in school. MF in preschool was positively associated with MF in school (*p* < 0.001) after adjusting for the confounding factors (R^2^ = 0.476, F = 8.090, *p* < 0.001). In girls, TPA in preschool was not associated with MF in school. MF in preschool was positively associated with MF in school (*p* = 0.001) after adjusting for the confounding factors (R^2^ = 0.429, F = 6.109, *p* = 0.001) (Table 2).

In boys, ST in preschool was not associated with MF in school. MF in preschool was positively associated with MF in school (*p* < 0.001) after adjusting for the confounding factors (R^2^ = 0.476, F = 8.090, *p* < 0.001). In girls, ST in preschool was not associated with MF in school. MF in preschool was positively associated with MF in school (*p* = 0.001) after adjusting for the confounding factors (R^2^ = 0.429, F = 6.109, *p* = 0.001) (Table 2).

## 4. Discussion

PA is a major modifiable determinant for increasing PF during growth in children [13], and there is a particularly paucity of longitudinal research. Hence, this study aimed to investigate whether VPA, MVPA, TPA, and ST at preschool age are associated with CRF and MF in the first grade of school among boys and girls. The main finding in the present study was that VPA, MVPA, TPA, and ST in preschool were unrelated to CRF and MF among boys and girls after adjustment for potential confounding factors, although in the unadjusted analysis, we found that, in boys, a higher VPA, MVPA, and TPA in preschool were associated with a higher MF in school, and a higher VPA in preschool was linked to a higher CRF in school among girls. Once adjusted for confounding factors, such as age, AWT, the corresponding PF item, and the highest parental education level at baseline, the observed associations between VPA, MVPA, and TPA in preschool and fitness later in school disappeared. We found, additionally, that a higher CRF and MF in preschool were associated with a higher CRF and MF, respectively, in school among boys in all adjusted models. Among girls, a higher MF in preschool was related to a higher MF in school.

The comparison of our results with previous findings shows some similarities [13,14,15,16,37,45] and some discrepancies [13,14,15,16,45]. Regarding the associations between PA and CRF, we found that, longitudinally, VPA, MVPA, and TPA were not associated with CRF after controlling for confounders, but earlier cross-sectional research showed that a higher MPA, VPA, and MVPA were correlated with a better CRF [13,14,15]. Specifically, a higher VPA and MVPA at 4–5 years of age were associated with better results in the 20 m shuttle run test after adjustment for gender, age, AWT, ST, and parental BMI and education level. While the results for MVPA were primarily due to VPA [14], Bürgi et al. [13] found that a higher MPA, VPA, and TPA were related to a higher CRF at 4–6-years of age after controlling for parental migrant status and education. Riso et al. [15] reported that MVPA, and particularly VPA, at 6–7 years of age were related to more laps in the 20 m shuttle run test after adjustment for age and gender (model 1) and for age, gender, organized sport participation, and parental education (model 2).

Regarding longitudinal research on this topic, Leppänen et al. [16] found that VPA and MVPA at the age of 4–5 years of age predicted a higher CRF one year later [16], which corresponded to their cross-sectional findings [14] and findings from our study before controlling for confounders among girls. In a longitudinal analysis, Bürgi et al. [13] demonstrated that VPA at the age of 4–6 years of age was related to the improvement in the 20 m shuttle run test during the 9-month follow-up after adjustment for baseline outcome parameters and parental sociocultural factors. Similar to results from the adjusted analysis of current study, they found no associations between TPA and CRF [13]. Additionally, Migueles et al. [37] reported that none of the movement behaviours (e.g., VPA, MPA, and ST) among 4-year-old children were associated with CRF and MF at 9 years of age directly after adjustment for gender, age, mother’s education, and group allocation. The compositional analysis found that more VPA relative to all the other behaviours at the age of 4 years was indirectly (via VPA at 9 years of age) associated with a better CRF at 9 years of age. While Potter et al. [45] demonstrated that a higher parent-reported PA (h/week) at 4.5 years of age predicted a better fitness composite score (incl. the 20 m shuttle run test performance, grip strength, vertical jump, sit-and-reach, and the inverse of waist circumference) at 3-year follow-up after adjustment for gender, follow-up age, and household income, the associations between PA and every single PF measure were insignificant [45]. To summarize, while some studies seem to relate PA with a greater CRF [13,14,15], the findings from the present and some other studies do not uniformly confirm this association [13,37,45]. Whether the analysis was conducted with a sample consisting both genders or separately among boys and girls could influence the results. Also, the age of the participants, the amount and content of PA they were engaged in, and as well the confounding factors included in the analysis could have played a role in the discrepancies. 

Concerning the associations between PA and muscular strength among preschool-aged children, we found that a higher VPA, MVPA, and TPA in the last preschool year were related to a higher MF among boys a year later in school before adjusting for confounding factors, although after adjustment, those associations disappeared. Among girls, PA was not related to MF neither in the unadjusted nor adjusted model. An earlier cross-sectional study showed that a higher VPA and MVPA among children aged 4–5 years were related to higher handgrip dynamometer and SLJ test results after adjustment for gender, age, AWT, ST, and parents’ BMI and education level [14]. At 6–7 years of age, a higher VPA and MVPA were related to a higher lower-limb strength, but not to upper-limb strength in either adjustment model [15]. Longitudinally, a higher VPA and MVPA at 4–5 years of age were associated with a higher lower-limb muscular strength at the one-year follow-up. A higher VPA was associated with a better handgrip strength before adjusting for confounding factors. However, after adjustments, the association disappeared [16].

To sum up, there is a paucity of studies that investigated the associations between PA and muscular strength in preschoolers, and the findings support a positive association with lower-limb strength, but the relationship with upper-limb strength is controversial, and the associations with compound MF have not been studied to date, which makes a direct comparison of our results with other studies more complicated. Perhaps, to achieve more pronounced and longer lasting effects from PA on PF, the intensity and total amount of PA should be increased in preschool institutions. Ek et al. [46] reported that preschool teachers perceived children’s VPA as low in their daily work and acknowledged that a PA of higher intensity (e.g., climbing, running) should be provided more often. The largest barriers for not offering more VPA were suboptimal facilities or time constraints [46]. Targeting such barriers could be important for future interventions to promote VPA in preschool-aged children [37]. Additionally, integrating daily and longer sessions of exercises that specifically target multiple aspects of fitness into the kindergarten schedule could be considered, as they have been shown to be effective in improving PF, especially among boys [47].

There is a negligible amount of research with mixed results on the associations between sedentary behaviours and CRF or muscular strength among preschool-aged children. Questionnaire-based ST at the age of 4.5 years was inversely associated with upper-limb strength, but not with vertical jump performance after controlling for gender, follow-up age, BMI, and household income [45]. Riso et al. [15] reported that, among 6–7-year-old children, a higher ST was related to a lower CRF in all adjusted models, and ST was inversely related to lower-limb strength after adjustment for age and gender, but not when an adjustment was made for age, gender, organized sport participation, and parental education. No association between time spent sedentary and grip strength was observed [15] that matched our longitudinal outcomes regarding MF. There were no associations between ST in preschool with CRF and MF in school among boys and girls in the current study. Leppänen et al. [14] found that ST at 4–5 years of age was associated with lower upper-limb strength, when PA was not adjusted, but after adjusting also for VPA, the association disappeared. No relations between ST with CRF and lower-limb strength were found, similar to the current study [14]. Corresponding with our results, ST at 4–5 years of age was not associated either with CRF and upper- or lower-limb strength a year later after controlling for several confounding factors [16]. 

The results of present investigation demonstrate no associations between PA measurements in preschool and PF measurements in the first grade of school after adjustments for confounding variables in boys and girls. However, preschool PF was frequently associated with PF in school, showing that, among boys, both CRF and MF values and, among girls, MF values track well from kindergarten to the first grade of school. Our results are similar to those of other studies that have found a fairly stable tracking of PF from childhood and/or adolescence to adulthood [48]. Thus, the CRF and MF parameters at preschool age can be used to characterize PF longitudinally at school in boys and MF parameters in girls, suggesting that building up higher PF levels should be targeted already from early childhood, as poor fitness is related with multiple chronic diseases [3,6,49].

We consider the strengths of our work to be the longitudinal design, objective assessment of PA, and the use of standardized PF tests. Yet, there are some limitations regarding the present study, like the inability of the accelerometer to capture activities performed in water and during cycling [50,51]. In addition, the relatively small sample size could have impacted our results, although our sample size was comparable to previous research in this area [52,53,54,55]. Finally, as this was an observational study, we cannot determine causal relationships.

## 5. Conclusions

In conclusion, while VPA, MVPA, TPA, and ST in preschool were not independently associated with CRF and MF among boys and girls in the first grade of school, a higher PF level in the last preschool year was often associated with a higher corresponding PF component in the first grade of school among boys and girls, suggesting that integrating exercises specifically designed to improve fitness into the daily PA schedule of preschoolers could be useful in order to enhance their PF.

## Figures and Tables

**Table 1:** Baseline data.

Variable	Boys	Girls	*p*
Age (years) ^1^	7 (1)	6 (1)	0.240
Height (cm)	127 (5.4)	124 (6.4)	**<0.001**
Weight (kg) ^1^	26 (6)	23.5 (6)	**0.010**
Physical activity			
VPA (min/day) ^1^	18 (12.7)	13.5 (12.3)	**0.042**
MVPA (min/day) ^1^	62.9 (29.4)	57.6 (29.2)	**0.003**
TPA (min/day)	382 (59.2)	359 (41.5)	**0.001**
ST (min/day) ^1^	382 (71.9)	400 (77.4)	0.237
Physical fitness test			
20 m shuttle run (laps) ^1^	17.5 (18)	17 (11)	0.383
Handgrip strength (kg) ^1^	11.5 (3)	9.9 (2.6)	**0.007**
Standing long jump (cm)	122 (18.7)	116 (15.3)	0.061
Muscular fitness z-score	−0.08 (0.6)	−0.09 (0.5)	0.797

Data are from the Student’s *t*-test or the Mann–Whitney U-test, or the chi-squared test for categorical variables, and are presented as means (standard deviations) and medians (interquartile ranges ^1^). VPA—vigorous physical activity; MVPA—moderate-to-vigorous physical activity; TPA—total physical activity; ST—sedentary time. Bold values denote *p* < 0.05.

**Table 2:** Multiple regression analysis demonstrating the associations of physical activity and sedentary time in preschool with physical fitness in school.

	Physical Fitness in School							
	CRF				Muscular Fitness			
	Boys (*n* = 41)		Girls (*n* = 36)		Boys (*n* = 41)		Girls (*n* = 36)	
Variables in Preschool	β	*p*	β	*p*	β	*p*	β	*p*
Unadjusted								
VPA	0.250	0.084	0.325	0.038	0.345	0.015	0.295	0.061
Adjusted								
VPA	0.130	0.367	0.139	0.449	−0.044	0.731	−0.011	0.947
Age	−0.136	0.349	−0.159	0.390	−0.209	0.104	0.140	0.357
Physical fitness *	0.560	**<0.001**	0.240	0.237	0.618	**<0.001**	0.642	**0.001**
Education	0.044	0.757	0.202	0.293	0.194	0.132	0.096	0.528
AWT	0.110	0.472	0.024	0.899	0.124	0.374	−0.091	0.569
Unadjusted								
MVPA	0.186	0.200	0.307	0.051	0.346	**0.015**	0.230	0.148
Adjusted								
MVPA	0.075	0.608	0.175	0.336	−0.078	0.547	−0.071	0.664
Age	−0.146	0.324	−0.164	0.371	−0.217	0.093	0.135	0.369
Physical fitness *	0.570	**<0.001**	0.245	0.214	0.628	**<0.001**	0.677	**0.001**
Education	0.057	0.692	0.197	0.301	0.198	0.121	0.088	0.566
AWT	0.119	0.443	0.045	0.810	0.132	0.346	−0.120	0.473
Unadjusted								
TPA	0.130	0.373	0.169	0.290	0.336	**0.018**	0.252	0.112
Adjusted								
TPA	0.139	0.352	0.134	0.451	−0.159	0.246	−0.063	0.694
Age	−0.131	0.372	−0.171	0.353	−0.242	0.064	0.133	0.376
Physical fitness *	0.586	**<0.001**	0.292	0.136	0.649	**<0.001**	0.674	**0.001**
Education	0.060	0.670	0.156	0.436	0.191	0.124	0.099	0.512
AWT	0.073	0.655	−0.018	0.921	0.173	0.231	−0.092	0.539
Unadjusted								
ST	0.111	0.448	−0.171	0.286	0.126	0.390	−0.038	0.812
Adjusted								
ST	−0.217	0.352	−0.378	0.451	0.246	0.246	0.182	0.694
Age	−0.131	0.372	−0.171	0.353	−0.242	0.064	0.133	0.376
Physical fitness *	0.586	**<0.001**	0.292	0.136	0.649	**<0.001**	0.674	**0.001**
Education	0.060	0.670	0.156	0.436	0.191	0.124	0.099	0.512
AWT	0.299	0.190	0.341	0.490	−0.082	0.707	−0.264	0.578

* Baseline physical fitness item (cardiorespiratory fitness or muscular fitness z-score) was entered into the model with the respective physical fitness item at follow-up. β—standardized regression coefficient; CRF—cardiorespiratory fitness; VPA—vigorous physical activity; AWT—accelerometer wear time; MVPA—moderate-to-vigorous physical activity; TPA—total physical activity; ST—sedentary time. Bold values denote *p* < 0.05.

## Data Availability

The datasets used in this study are available from the corresponding author upon reasonable request due to privacy and ethical reasons.
